# Construction of a novel cuproptosis-related gene signature for predicting prognosis and estimating tumor immune microenvironment status in papillary thyroid carcinoma

**DOI:** 10.1186/s12885-022-10175-5

**Published:** 2022-11-04

**Authors:** Lidong Wang, Baiyu Yao, Jiapeng Yang, Zhong Tian, Jingni He

**Affiliations:** Department of General Surgery, Shengjing Hospital of China Medical University, Shenyang, Liaoning Province China

**Keywords:** Cuproptosis-related genes, Papillary thyroid carcinoma, Prognosis, Risk model, Tumor immune microenvironment

## Abstract

**Background:**

Cuproptosis, a new form of programmed cell death, has been recently reported to be closely related to tumor progression. However, the significance of cuproptosis-related genes (CRGs) in papillary thyroid carcinoma (PTC) is still unclear. Therefore, this study aimed to investigate the role of the CRG signature in prognosis prediction and immunotherapeutic effect estimation in patients with PTC.

**Methods:**

RNA-seq data and the corresponding clinical information of patients with PTC were obtained from the Cancer Genome Atlas (TCGA) and Gene Expression Omnibus (GEO) databases. Comprehensive analyses, namely, consensus clustering, immune analyses, functional enrichment, least absolute shrinkage and selection operator-multivariate Cox regression, and nomogram analysis, were performed to identify new molecular subgroups, determine the tumor immune microenvironment (TIME) status of the identified subgroups, and construct a clinical model. Independent verification cohort data and quantitative real-time polymerase chain reaction (qPCR) was performed to validate the expression of specific prognosis-related and differentially expressed CRGs (P-DECRGs).

**Results:**

In the TCGA database, 476 patients with PTC who had complete clinical and follow-up information were included. Among 135 CRGs, 21 were identified as P-DECRGs. Two molecular subgroups with significantly different disease-free survival and TIME statuses were identified based on these 21 P-DECRGs. The differentially expressed genes between the two subgroups were mainly associated with immune regulation. The risk model and nomogram were constructed based on four specific P-DECRGs and validated as accurate prognostic predictions and TIME status estimation for PTC by TCGA and GEO verification cohorts. Finally, the qPCR results of 20 PTC and paracancerous thyroid tissues validated those in the TCGA database.

**Conclusions:**

Four specific P-DECRGs in PTC were identified, and a clinical model based on them was established, which may be helpful for individualized immunotherapeutic strategies and prognostic prediction in patients with PTC.

**Supplementary Information:**

The online version contains supplementary material available at 10.1186/s12885-022-10175-5.

## Background

The incidence of thyroid carcinoma, the most common endocrine malignancy, has continuously and rapidly increased worldwide in recent decades [1]. However, thyroid carcinoma mortality remains relatively stable at low levels [2]. In 2021, the estimated numbers of new thyroid cancer cases and deaths in the United States were 44,280 and 2,200, respectively [3]. Similar thyroid carcinoma trends have been reported in China [4]. The most frequent histological type of thyroid carcinoma is papillary thyroid carcinoma (PTC), which accounts for approximately 90% of all cases [5]. Most PTCs are well-differentiated, indolent, and have excellent prognoses when conventional treatments are implemented. However, several clinicopathological features, including age, tumor diameter, extrathyroidal extension, and cervical lymph node and distant organ metastases, are considered significantly unfavorable prognostic factors and result in a relatively high recurrence rate [6]. Our institution has reported the risk factors associated with cervical lymph node metastasis of PTC and provided some clinical suggestions regarding prophylactic central lymph node dissection [7]. To date, the molecular mechanisms of recurrence and metastasis in PTC are not fully understood. Therefore, a deeper investigation of potential therapeutic targets and more efficient prognostic models for PTC are of great clinical significance, especially for facilitating personalized treatment strategies.

Copper is an indispensable trace element that plays an essential role in various biological processes. Dysregulation of copper homeostasis, i.e., the abnormal alteration of intracellular copper levels, may trigger cytotoxicity and is considered a novel hallmark of malignant tumor progression [8]. Recently, cuproptosis has been identified as a unique type of cell death related to copper homeostasis disorder, proving that the dysregulation of copper homeostasis plays a pivotal role in tumor development and progression. Additionally, several genes involved in cuproptosis have been identified [9]. However, the role of cuproptosis-related genes (CRGs) in PTC remains poorly understood.

In the present study, we aimed to comprehensively investigate the correlations of CRGs with different survival statuses of patients with PTC, as well as the underlying molecular mechanisms. Our study highlighted the regulatory functions of CRGs in PTC progression. Moreover, the results shed light on novel strategies to predict prognosis and lay a foundation for individual-based therapeutic application in patients with PTC.

## Materials and methods

### Data collection

Gene expression profiles and clinical information on thyroid carcinoma from The Cancer Genome Atlas (TCGA) database were obtained from the cBioPortal for Cancer Genomics, including 502 tumor and 58 non-tumor cases [10, 11]. We enrolled patients with PTC who had complete information on age, sex, tumor/lymph node/metastasis (TNM) staging system, follow-up, and survival status (disease-free survival [DFS]). The clinicopathological features of PTC samples from TCGA are presented in Table 1. Samples acquired from seven Gene Expression Omnibus (GEO) databases (GSE3678, GSE6004, GSE29265, GSE33630, GSE35570, GSE53157, and GSE60542) were integrated and defined as an independent verification cohort, including 196 PTC and 160 normal thyroid cases. The matched mapping information of GeneSymbol and ENSG_ID was extracted from the gff3 files and downloaded from GENCODE. The median was obtained when multiple matches existed, and finally, the converted expression spectrum (convert_exp.txt) was obtained. To deal with missing data completion, both genes and cases with unavailable (NA) ratios greater than 50% were removed. To standardize the data, we performed a log2 (X + 1) conversion. Datasets of cuproptosis-related genes (CRGs) were obtained from the Molecular Signatures Database (MSigDB) and a previously published cuproptosis-related study [9, 12].

**Table 1:** Clinical characteristics of patients with PTC obtained from the TCGA database in the present study

Clinical characteristics	Total (*n* = 476)	Training cohort (*n* = 238)	Verification cohort (*n* = 238)	χ2	*P*
Gender				0.271	0.603
Female	351	178	173		
Male	125	60	65		
Age				1.402	0.236
< 55	326	157	169		
≥ 55	150	81	69		
T stage				0.998	0.910
T1	137	64	73		
T2	160	84	76		
T3	159	80	79		
T4	18	9	9		
Tx	2	1	1		
N stage				0.416	0.812
N0	221	114	107		
N1	208	101	107		
Nx	47	23	24		
M stage				0.144	0.959
M0	267	132	135		
M1	8	4	4		
Mx	201	102	99		
Pathologic stage				2.932	0.402
I	273	128	145		
II	49	28	21		
III	104	54	50		
IV	50	28	22		
Disease free status				0.096	0.756
Yes	430	216	214		
No	46	22	24		

*PTC* papillary thyroid carcinoma, *TCGA* the Cancer Genome Atlas, *T* tumor, *N* lymph node, *M* metastasis

### Tissue samples

A total of 20 patients who underwent thyroid surgery and were diagnosed with PTC by postoperative pathology between January 2022 and May 2022 at Shengjing Hospital of China Medical University were included. The inclusion criteria for selecting patients were age > 18 years, a postoperative pathological diagnosis of PTC, and complete patient clinical information. In addition, the exclusion criteria were other treatments, such as radiotherapy, chemotherapy, or immunotherapy, received before surgery; patients with serious systemic diseases, such as severe heart, liver, or kidney diseases and other malignant tumors; women in the gestational and lactational period; patients taking drugs with potential interference; patients who refused or were unable to sign informed consent; other histological types of thyroid carcinoma combined with PTC in postoperative pathological diagnosis. PTC and paracancerous thyroid tissues at least 2 cm away from PTC areas and confirmed as being without PTC cells by two pathologists were collected following protocols approved by the Ethics Committee of Shengjing Hospital of China Medical University. Written informed consent was obtained from all patients enrolled in this study.

### Identification of molecular subgroups related to CRGs

Differentially expressed CRGs (DECRGs) in PTC and non-tumor cases from the TCGA database were determined using the Wilcoxon rank-sum test. The association between these DECRGs and PTC prognosis was compared using univariate Cox regression analysis. Next, cluster analysis of these prognosis-related DECRGs (P-DECRGs) was performed by “ConsensusClusterPlus” using agglomerative pam clustering with 1-Pearson correlation distances and resampling 80% of the samples for 10 repetitions [13]. The optimal number of clusters was determined using an empirical cumulative distribution function plot. The R software package “stats” (version 3.6.0) was used for principal component analysis (PCA). Briefly, we first determined the Z-score on the expression spectrum and then used the “prcomp” function for dimension reduction analysis to obtain the reduced dimension matrix.

### Kaplan–Meier plot

We used the “survfit” function of the R software package “survival” to analyze the prognostic differences between different groups of samples and the log-rank test method to evaluate the significance.

### Functional analyses

Differentially expressed genes (DEGs) between molecular subgroups related to P-DECRGs were identified using the R package “linear models for microarray data (Limma, version 3.40.6)” [14]. Specifically, for the expression profile data set we obtained, we used the “lmFit” function to perform multiple linear regression. Further, we used the “eBays” function to compute moderated *t*- and F-statistics and log-odds of differential expression by empirical Bayes moderation of the standard errors towards a common value. Finally, we obtained the significance of the differences of each gene. For Gene Ontology (GO) and Kyoto Encyclopedia of Genes and Genomes (KEGG) enrichment analysis, we downloaded the “c5.go.v7.4.symbols.gmt” subset from the molecular signatures database and used the KEGG rest API to obtain the latest gene annotations for KEGG pathway analysis [15–17]. Considering these as the background, we mapped the genes into the background set and used the R software package “clusterProfiler” (version 3.14.3) for enrichment analysis of the gene set. The minimum and maximum gene sets were set to 5 and 5,000, respectively. *P* < 0.05, and false discovery rate (FDR) < 0.1 were considered statistically significant.

### Immune analyses

The stromal and immune scores and tumor purity, defined as the quantitative proportions of immune and stromal components and malignant cells in tumor samples in different groups, were calculated using the estimation of stromal and immune cells in malignant tumor tissues using expression data (ESTIMATE) algorithm [18]. The scores of four different immunophenotypes, including major histocompatibility complex (MHC) molecules, effector cells, immunosuppressive cells, and immune checkpoints, were calculated using the immunophenoscore (IPS) algorithm [19]. The human immune cell subsets were calculated using the cell type identification by estimating relative subsets of RNA transcripts (CIBERSORT) and the quantification of the tumor immune contexture from human RNA-seq data (quanTIseq) algorithms [20, 21]. The responses of patients with PTC to immune checkpoint blockade (ICB) therapy were predicted using the immune cell abundance identifier (ImmuCellAI) algorithm [22].

### Establishment and verification of the risk model related to specific P-DECRGs

The PTC sample dataset from the TCGA database was randomly divided into training and verification cohorts (1:1). We used the R software package “glmnet” to integrate survival time and status and gene expression data and used the least absolute shrinkage and selection operator (LASSO)-Cox method for regression analysis. In addition, a tenfold cross-validation was performed to obtain the minimum lambda, which was defined as the optimal value. In addition, a risk model related to specific P-DECRGs was established using a multivariate Cox regression analysis. We used the R software package “survival” to integrate survival time and status and gene expression data and the Cox method to evaluate the prognostic significance of each gene. Patients with PTC were divided into high- and low-risk groups according to the medium value of the risk score. Next, we used the R software package “pROC” (version 1.17.0.1) to perform a receiver operating characteristic (ROC) analysis to assess the predictive efficiency of the risk model. Specifically, we obtained patient follow-up times and risk scores and used the pROC function to perform ROC analysis at multiple time points and the CI function of pROC to evaluate the area under the curve (AUC) and confidence interval to obtain the final AUC results.

### Establishment and verification of a nomogram scoring system related to risk score

By integrating the DFS time, survival status, clinicopathological features, and risk score data, a predictive nomogram was established using the package “rms,” and the prognostic significance of these characteristics was evaluated [23]. In the nomogram scoring system, each variable was matched to a score. The total score of each sample was the sum of all variable scores. Time-dependent ROC curves and calibration plots were used to assess the nomogram-scoring system.

### RNA extraction and quantitative real-time polymerase chain reaction (qPCR)

Total RNA was extracted using an RNAiso Plus Kit (Takara, Dalian, China) according to the manufacturer’s instructions. After reverse transcription, cDNA was synthesized, and qPCR analysis was performed using SYBR Premix Real-time PCR Reagent (Takara) in a Roche LightCycler 480 II system (Roche Diagnostics Corporation, Indianapolis, IN, USA) according to the manufacturer’s protocol. The internal control was glyceraldehyde-3-phosphate dehydrogenase (*GAPDH*). The primer sequences of the selected target genes and internal controls are listed in Table 2. The target gene expression levels were calculated using the 2 ^–ΔΔCt^ method, similar to that in one of our previously reported studies [24].

**Table 2:** Primer sequences of genes used in this study

Gene	Primer sequence	Product length (bp)
*FDX1*	Forward: 5′-CTTTGGTGCATGTGAGGGAA-3′ Reverse: 5′-GCATCAGCCACTGTTTCAGG-3′	216
*P2RX4*	Forward: 5′-TGGCGGATTATGTGATACCAGC-3′ Reverse: 5′-GTCGCATCTGGAATCTCGGG-3′	112
*CDK1*	Forward: 5′-CCCTTTAGCGCGGATCTACC-3′ Reverse: 5′-CATGGCTACCACTTGACCTGT-3′	133
*MAP1LC3A*	Forward: 5′-CCAGCAAAATCCCGGTGA-3′ Reverse: 5′-TGGTCCGGGACCAAAAACT-3′	88
*GAPDH*	Forward: 5′-GCACCGTCAAGGCTGAGAAC-3′ Reverse: 5′-TGGTGAAGACGCCAGTGGA-3′	138

### Statistical analyses

Enumeration data are presented as numbers/percentages, whereas measurement data are presented as mean ± standard deviation (SD). Student’s *t*-test was used for the statistical analysis of two groups, and one-way ANOVA was used for three or more groups. Statistical analyses were performed using R software (version 4.0.3; R Foundation for Statistical Computing, Vienna, Austria). Statistical significance was set at *P* < 0.05.

## Results

### Identification of P-DECRGs in PTC

**Fig. 1:**
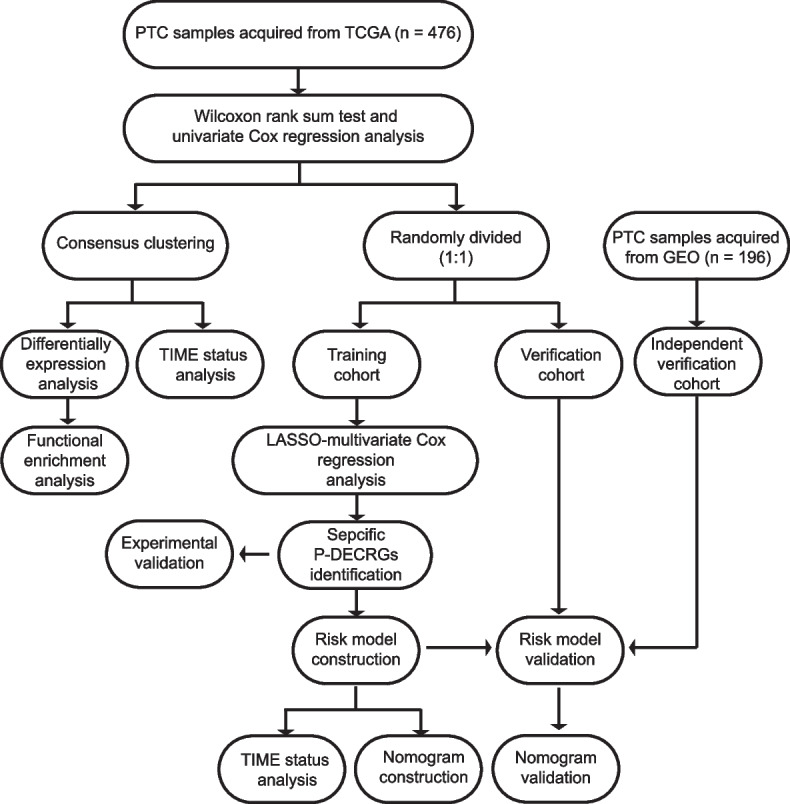
Data analysis flow chart. PTC, papillary thyroid carcinoma; TCGA, the Cancer Genome Atlas; GEO, Gene Expression Omnibus; LASSO, least absolute shrinkage and selection operator; P-DECRGs, prognostic associated and differentially expressed cuproptosis-related genes; TIME, tumor immune microenvironment

A flow chart of the data analysis in the present study is shown in Fig. 1. Among the 502 patients with thyroid carcinoma from the TCGA dataset, 476 patients with PTC who had complete clinical and survival information were selected. To investigate the potential roles of CRGs in PTC, 135 CRGs were obtained from 12 gene sets in MSigDB and a previously published cuproptosis-related study (Additional file 1: Table S1). We matched these 135 genes with the RNA-seq data of 476 PTC and 58 non-tumor samples from the TCGA database. Compared with non-tumor samples, 107 CRGs were differentially expressed in PTC. Among these 107 genes, only 21 P-DECRGs, generated from the univariable Cox analysis results, were selected for subsequent analysis (Additional file 2: Table S2).

### Identification of two molecular subtypes based on P-DECRGs in PTC

**Fig. 2:**
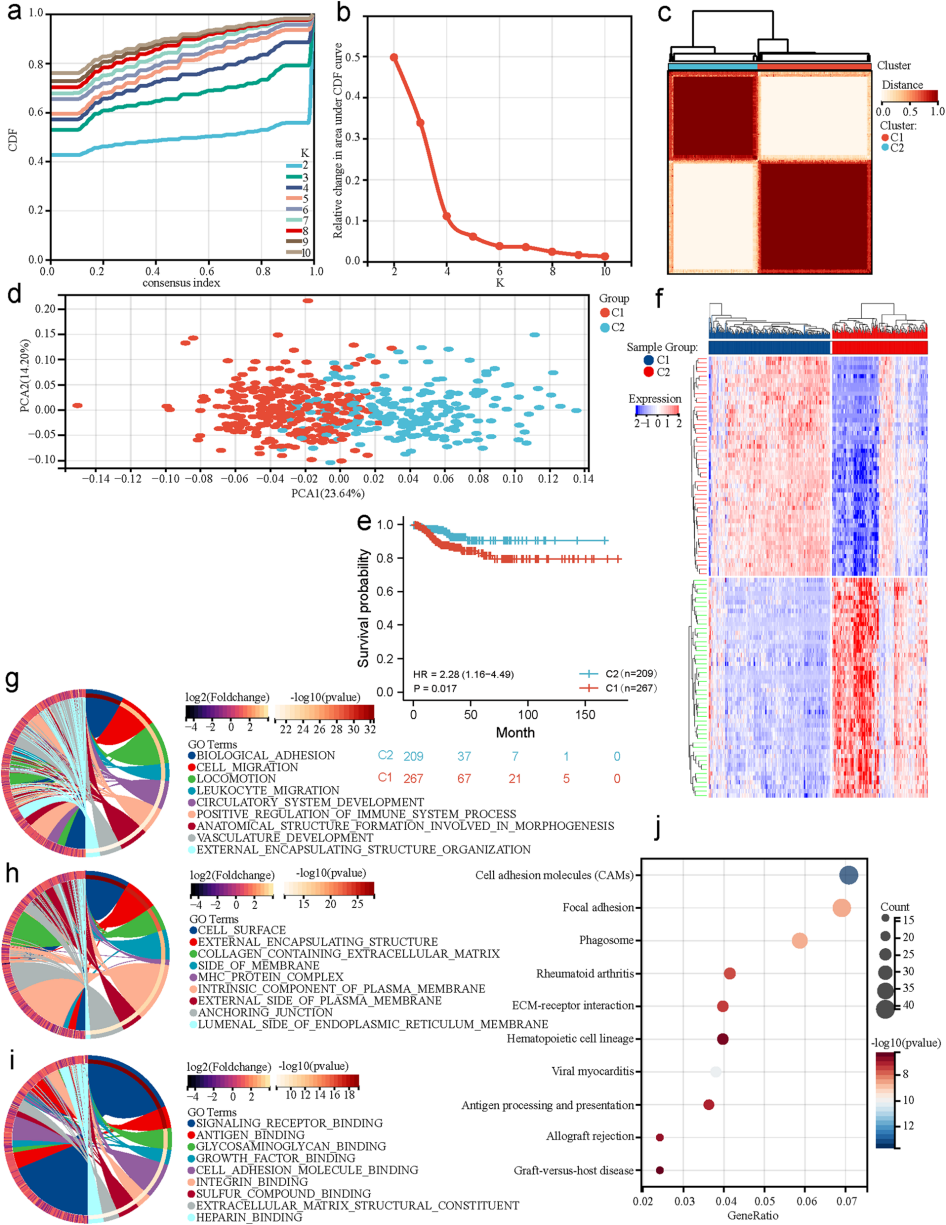
Identification of the two molecular subtypes based on P-DECRGs through consensus clustering in PTC. **a** CDF values and (**b**) Relative changes in the area under the CDF curve corresponding to different consensus matrices for K = from 2 to 10. **c** The consensus clustering matrix defining two clusters (K = 2) and their correlation area visualized using a heatmap. **d** Different transcriptional expressions between C1 and C2 analyzed by PCA. **e** A remarkable difference in DFS was observed between C1 and C2, which were visualized using a Kaplan–Meier plot. **f** The heatmap, **g** GO biological processes, **h** Cellular components, and (**i**) Molecular function enrichment analysis results of DEGs between C1 and C2 visualized using Circos. **j** KEGG enrichment analysis results of DEGs between C1 and C2 visualized using a bubble chart. PTC, papillary thyroid carcinoma; P-DECRGs, prognostic associated and differentially expressed cuproptosis-related genes; C1, cluster1; C2, cluster2; CDF, cumulative distribution function; PCA, principal components analysis; HR, hazard ratio; DEGs, differentially expressed genes; GO, Gene Ontology; KEGG, Kyoto Encyclopedia of Genes and Genomes

The consensus clustering approach was used to divide the 476 patients with PTC into subgroups based on the 21 P-DECRGs. The clustering results showed that optimal clustering stability was identified when K = 2 (Fig. 2a and b). A total of 267 patients with PTC were clustered into cluster 1 (C1), and the remaining 209 patients with PTC were clustered into C2 (Fig. 2c). The PCA results revealed significant differences in the expression levels of P-DECRGs between the two molecular subtypes (Fig. 2d). Moreover, patients in C2 had better DFS than those in C1 (*P* < 0.05; Fig. 2e). These results demonstrate that the 21 P-DECRGs could divide patients with PTC into two molecular subtypes with different prognostic results.

### Functional enrichment of differentially expressed genes between the two P-DECRG-based molecular subtypes

To explore the underlying signaling mechanisms between the two P-DECRG-based molecular subtypes, the DEGs between these two clusters were identified. Compared with C2, the results showed that a total of 1,351 DEGs were identified, of which 775 genes were upregulated, and 576 genes were downregulated in C1 (Fig. 2f). GO biological process enrichment analysis revealed that the DEGs were enriched in biological adhesion, cell migration, locomotion, leukocyte migration, and the positive regulation of immune system processes (Fig. 2g). GO cellular component enrichment analysis revealed that the DEGs were enriched in the cell surface, external encapsulating structure, collagen-containing extracellular matrix, side of membrane, and MHC protein complex (Fig. 2h). GO molecular function enrichment analysis revealed that the DEGs were enriched in signaling receptor, antigen, glycosaminoglycan, growth factor, and cell adhesion molecule binding (Fig. 2i). KEGG enrichment analysis revealed that the DEGs were enriched in many pathways, including cell adhesion molecules, viral myocarditis, phagosome, focal adhesion, rheumatoid arthritis, and extracellular matrix (ECM)-receptor interaction (Fig. 2j). All these results demonstrated that the divided molecular subtypes were correlated with the regulation of the immune system process, which may be involved in the DFS of patients with PTC.

### Different tumor immune microenvironment (TIME) statuses in the two P-DECRG-based molecular subtypes

**Fig. 3:**
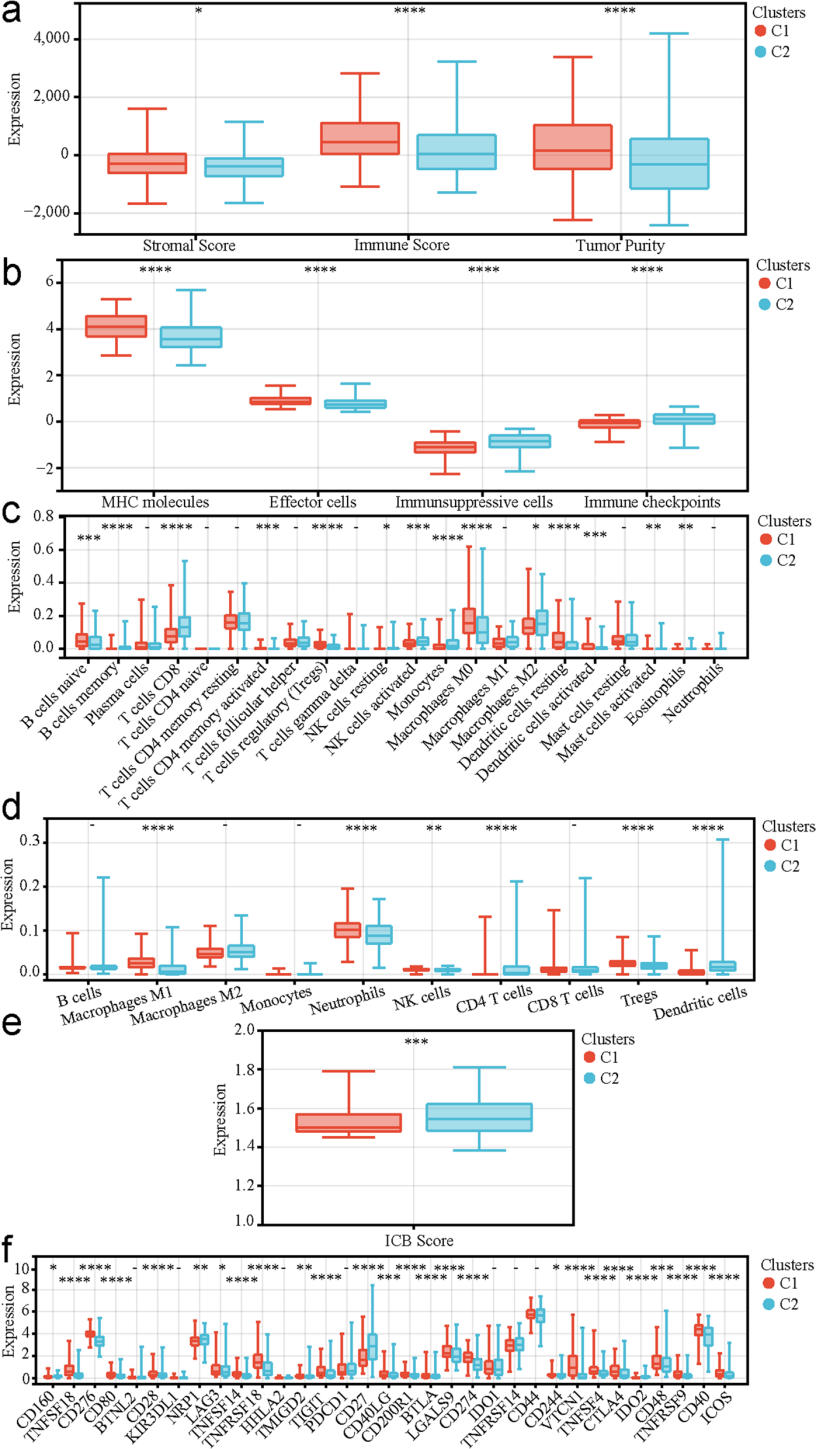
Differences in tumor immune microenvironments between C1 and C2. **a** The differences in stromal and immune scores and tumor purity between C1 and C2 based on the ESTIMATE algorithm. **b** The differences in MHC molecules, effector cells, immunosuppressive cells, and immune checkpoints between C1 and C2 based on the IPS algorithm. **c** The differences in the 22 types of immune cell infiltrating abundances between C1 and C2 based on the CIBERSORT algorithm. **d** The differences of immune cell infiltrating abundances between C1 and C2 based on the quanTIseq algorithm. **e** The differences in ICB therapy scores between C1 and C2 based on the ImmuCellAI algorithm. **f** The differences in the expression levels of the 33 known types of immune checkpoint molecules between C1 and C2. C1, cluster1; C2, cluster2; MHC, major histocompatibility complex; ICB, immune checkpoint blockade. * *P* < 0.05, ** *P* < 0.01, *** *P* < 0.001, **** *P* < 0.0001

To explore the differences in TIME status between the two molecular subtypes, we performed multiple immune analyses. Compared with those in C1, the ESTIMATE algorithm results revealed that patients with PTC in C2 had significantly lower stromal scores (*P* < 0.05), immune scores (*P* < 0.0001), and tumor purity (*P* < 0.0001; Fig. 3a). The IPS algorithm results showed that patients with PTC in C2 had significantly lower MHC molecules (*P* < 0.0001) and effector cells (*P* < 0.0001), and higher immunosuppressive cells (*P* < 0.0001) and immune checkpoints (*P* < 0.0001; Fig. 3b) compared to those in C1. The CIBERSORT algorithm results indicated that the infiltration levels of naive B cells, CD4 memory activated T cells, regulatory T cells (Tregs), M0 macrophages, resting dendritic cells, activated dendritic cells, and activated mast cells were significantly higher in C1 than those in C2. In contrast, memory B cells, CD8 T cells, resting NK cells, activated NK cells, monocytes, M2 macrophages, and eosinophils had significantly lower infiltration in C1 than those in C2 (Fig. 3c). The quanTIseq algorithm results showed that the infiltration levels of M1 macrophages, neutrophils, NK cells, and Tregs were significantly higher in C1 than those in C2, while CD4 T cells and dendritic cells had significantly lower infiltration in C1 compared to those in C2 (Fig. 3d). The results of the ImmuCellAI algorithm revealed that ICB therapy scores were significantly lower in C1 than those in C2 (Fig. 3e). In addition, the investigation of the expression differences of 33 known types of immune checkpoint molecules showed that 26 types were differentially expressed between the two molecular subtypes (Fig. 3f). All these results demonstrated significant differences in TIME status between the C1 and C2 subtypes.

### Establishment of a risk model based on specific P-DECRGs in the training cohort

**Fig. 4:**
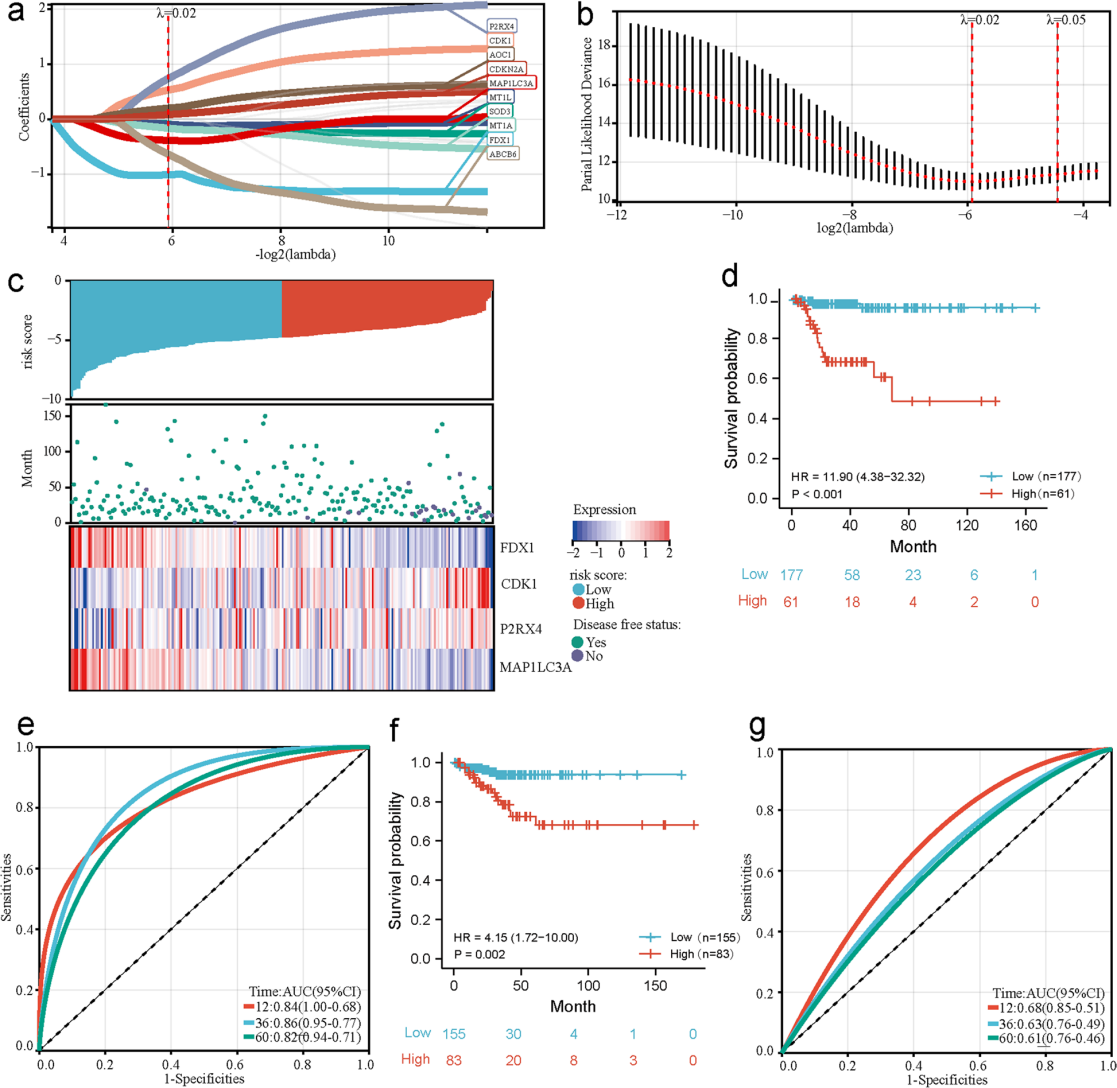
Establishment and validation of risk model based on specific P-DECRGs. **a** Coefficients and (**b**) Partial likelihood deviances corresponding to different lambda values in the LASSO regression analysis. **c** The correlation between risk score and DFS visualized using ranked bar (upper part) and scatter plots (middle part). The correlation between the expression of the four specific P-DECRGs and risk score visualized using a heatmap (lower part). **d** The significant difference in DFS between high- and low-risk groups observed and visualized using a Kaplan–Meier plot. **e** The sensitivities and specificities of 1-, 3-, and 5-year DFS predictions based on the risk model; these are visualized using ROC curves. **f** The significant difference in DFS between high- and low-risk groups in the verification cohort observed and visualized using a Kaplan–Meier plot. **g** The sensitivities and specificities of 1-, 3-, and 5-year DFS predictions based on the risk model in the verification cohort visualized using ROC curves. P-DECRGs, prognostic associated and differentially expressed cuproptosis-related genes; LASSO, least absolute shrinkage and selection operator; DFS, disease-free survival; ROC, receiver operating characteristic; AUC, area under the curve; HR, hazard ratio

To assess the prognostic value of P-DECRGs in PTC, a risk signature model based on P-DECRGs in the training cohort was constructed. First, the potential P-DECRGs for establishing the risk model were screened using LASSO regression analysis. The results showed that the optimal lambda value was 0.0164684245862992, and 10 genes were filtered (Fig. 4a and b). In addition, multivariate Cox analysis was performed based on the genes generated from LASSO analysis, and four specific P-DECRGs of PTC were identified: Ferredoxin 1 (*FDX1*), Cyclin Dependent Kinase 1 (*CDK1*), Purinergic Receptor P2X 4 (*P2RX4*), and Microtubule Associated Protein 1 Light Chain 3 Alpha (*MAP1LC3A*). A risk model of these four specific genes was constructed to calculate the risk score as follows:

Risk score = -2.011 × the expression level of *FDX1* + 1.139 × the expression level of *CDK1* + 1.662 × the expression level of *P2RX4* − 0.948 × the expression level of *MAP1LC3A*.(1).

Patients with PTC in the training cohort were successfully classified into high- and low-risk groups based on the medium risk score of the established risk model. We analyzed the relationship between different risk scores and patient follow-up times, events, and changes in the expression of various genes. It was observed that, with an increase in risk scores, the survival rate of patients decreased, and the expression of *FDX1* and *MAP1LC3A* showed a downward trend. However, the expression of *CDK1* and *P2RX4* was upregulated with an increase in risk score (Fig. 4c). Patients with PTC in the high-risk group had a worse DFS than those in the low-risk group (Fig. 4d). The AUCs of the ROC curve for 1-, 3-, and 5-year survival were 0.84, 0.86, and 0.82, respectively, according to the results of time-dependent ROC analysis in the training cohort (Fig. 4e). These results suggest that the constructed risk model based on the four specific P-DECRGs showed considerable potential for predicting the DFS of patients with PTC.

### Validation of the risk model based on specific P-DECRGs in the PTC verification cohort of PTC

To validate the constructed risk model based on the four specific P-DECRGs, patients with PTC in the TCGA verification cohort and those in the independent GEO verification cohort were also divided into high- or low-risk groups. Survival analysis revealed that patients from the TCGA verification cohort in the low-risk group had a better prognosis than those in the high-risk group (Fig. 4f). The AUC of the ROC curve for 1-, 3-, and 5-year survival was 0.68, 0.63, and 0.61, respectively, which indicated that the constructed risk model exhibited predictive capacity, according to the results of time-dependent ROC analysis in the verification cohort (Fig. 4g). These results validated that the risk model based on the four specific P-DECRGs was well established and associated with predicting the DFS of patients with PTC in the verification cohort.

### Different TIME statuses in the two risk groups based on the constructed risk model

**Fig. 5:**
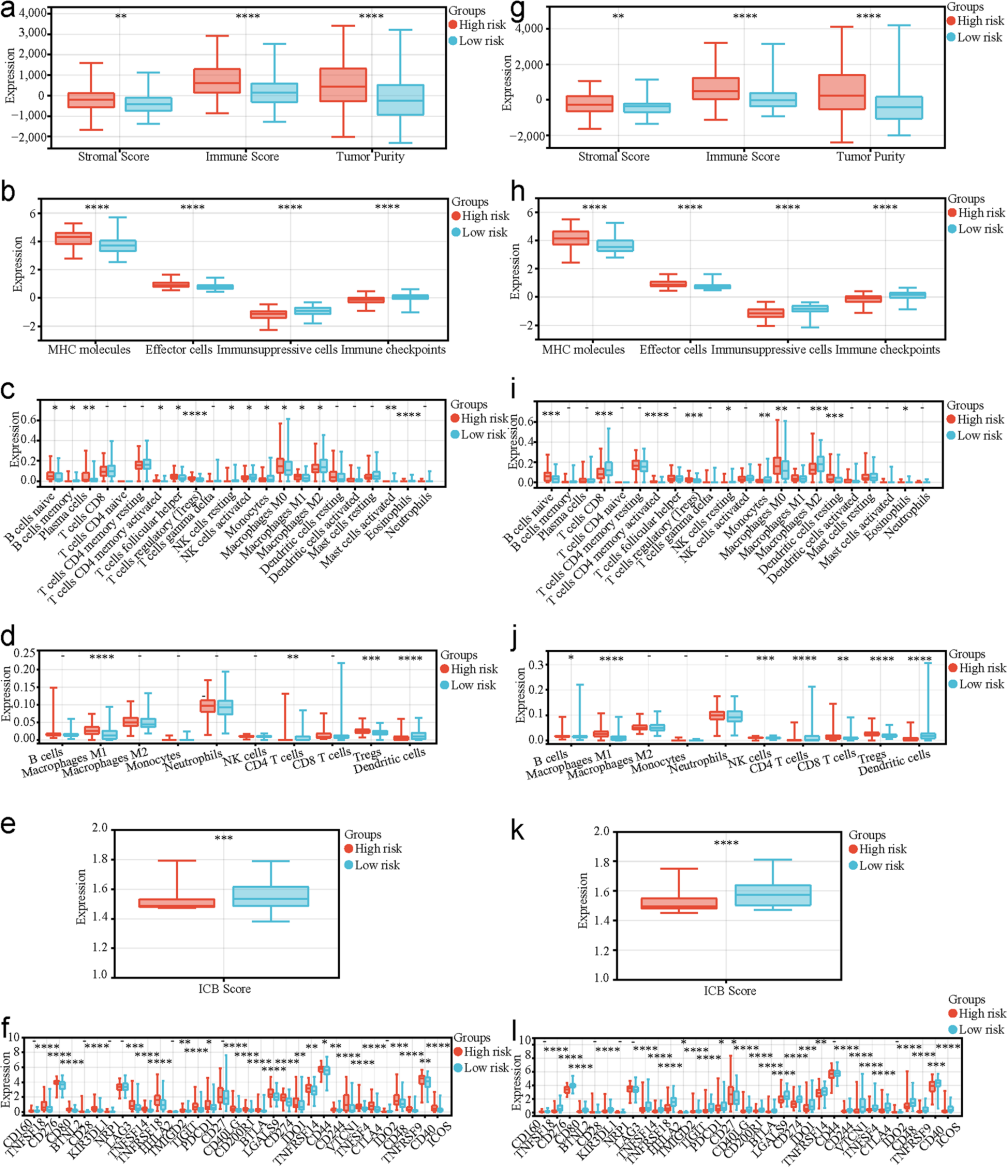
Different TIME statuses between high- and low-risk groups of risk model based on specific P-DECRGs in the training and TCGA verification cohort. The differences in stromal and immune scores and tumor purity between high- and low-risk groups (**a**) in the training cohort and (**g**) in the TCGA verification cohort based on the ESTIMATE algorithm. The differences in MHC molecules, effector cells, immunosuppressive cells, and immune checkpoints between high- and low-risk groups (**b**) in the training cohort and (**h**) in the TCGA verification cohort based on the IPS algorithm. The differences in the 22 types of immune cell infiltrating abundances between high- and low-risk groups (**c**) in the training cohort and (**i**) in the TCGA verification cohort based on the CIBERSORT algorithm. The differences of immune cell infiltrating abundances between high- and low-risk groups (**d**) in the training cohort and (**j**) in the TCGA verification cohort based on the quanTIseq algorithm. The differences in ICB therapy scores between high- and low-risk groups (**e**) in the training cohort and (**k**) in the TCGA verification cohort based on the ImmuCellAI algorithm. The differences in the expression levels of the 33 known types of immune checkpoint molecules between high- and low-risk groups (**f**) in the training cohort and (l) in the TCGA verification cohort. PTC, papillary thyroid carcinoma; P-DECRGs, prognostic associated and differentially expressed cuproptosis-related genes; TIME, tumor immune microenvironment; DFS, disease-free survival; ROC, receiver operating characteristic; AUC, area under the curve; MHC, major histocompatibility complex; ICB, immune checkpoint blockade; TCGA, the Cancer Genome Atlas. * *P* < 0.05, ** *P* < 0.01, *** *P* < 0.001, **** *P* < 0.0001

**Fig. 6:**
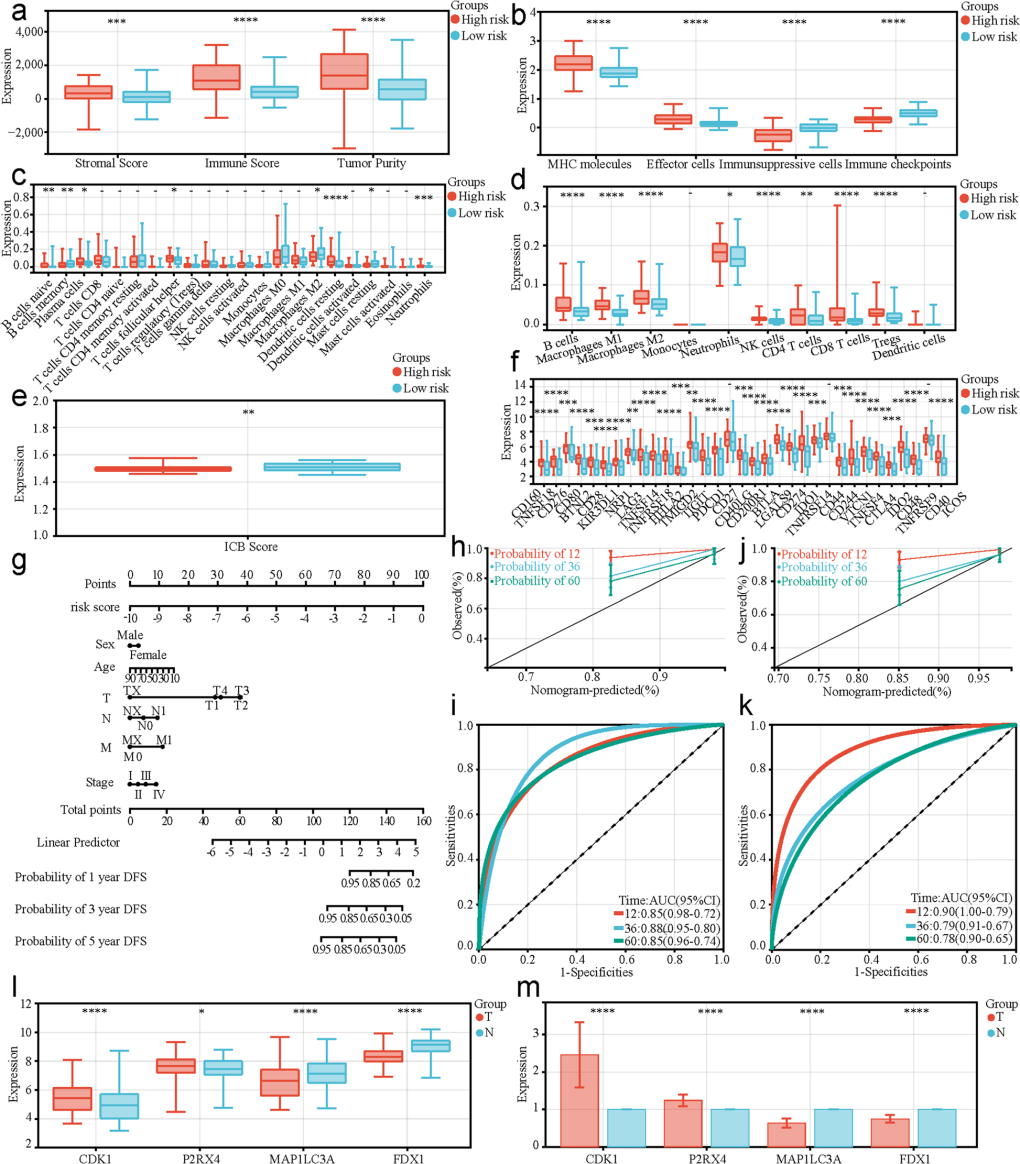
Construction and calibration of a nomogram integrating the risk model and experimental validation of the four specific P-DECRGs. The differences of TIME statues based on (**a**) ESTIMATE, **b** IPS, **c** CIBERSORT, **d** quanTIseq, **e** ImmuCellAI algorithms and (**f**) the expression levels of the 33 known types of immune checkpoint molecules between high- and low-risk groups in the independent GEO verification cohort. **g** A nomogram for predicting the 1-, 3-, and 5-year DFS of patients with PTC in the training cohort. Calibration curves of the nomogram for 1-, 3-, and 5-year DFS predictions of patients with PTC **h** in the training cohort and (**j**) in the TCGA verification cohort. The sensitivities and specificities of 1-, 3-, and 5-year DFS predictions of patients with PTC (**i**) in the training cohort and (**k**) in the TCGA verification cohort based on the nomogram and visualized using ROC curves. **f** Relative mRNA expression of the four specific P-DECRGs in PTC and non-tumor thyroid tissues from (**l**) the independent GEO verification cohort and (**m**) 20 patients. PTC, papillary thyroid carcinoma; P-DECRGs, prognostic associated and differentially expressed cuproptosis-related genes; ROC, receiver operating characteristic; AUC, area under the curve; TIME, tumor immune microenvironment; DFS, disease-free survival; ROC, receiver operating characteristic; AUC, area under the curve; MHC, major histocompatibility complex; ICB, immune checkpoint blockade; TCGA, the Cancer Genome Atlas; GEO, Gene Expression Omnibus. * *P* < 0.05, **** *P* < 0.0001

To explore the association between risk score and TIME status, we performed multiple immune analyses. In the training cohort, the ESTIMATE algorithm results revealed that patients with PTC in the high-risk group had significantly higher stromal scores (*P* < 0.01), immune scores (*P* < 0.0001), and tumor purity (*P* < 0.0001) than those in the low-risk group (Fig. 5a). The IPS algorithm results showed that patients with PTC in the high-risk group had significantly higher MHC molecules (*P* < 0.0001) and effector cells (*P* < 0.0001), and lower immunosuppressive cells (*P* < 0.0001) and immune checkpoints (*P* < 0.0001) than those in the low-risk group (Fig. 5b). The CIBERSORT algorithm indicated that the infiltration levels of naive B cells, plasma cells, CD4 memory activated T cells, follicular helper T cells, Tregs, M0 macrophages, M1 macrophages, eosinophils, and neutrophils were significantly higher in the high-risk group than those in the low-risk group, while memory B cells, resting NK cells, activated NK cells, monocytes, M2 macrophages, and activated mast cells had significantly lower infiltration in the high-risk group than those in the low-risk group (Fig. 5c). The quanTIseq algorithm results showed that the infiltration levels of M1 macrophages and Tregs were significantly higher in the high-risk group than those in the low-risk group, while CD4 T cells and dendritic cells had significantly lower infiltration in the high-risk group compared to those in the low-risk group (Fig. 5d). The ImmuCellAI algorithm results revealed that ICB therapy scores were significantly lower in the high-risk group than those in the low-risk group (Fig. 5e). Among the 33 known types of immune checkpoint molecules, 26 were differentially expressed between the two risk groups (Fig. 5f). Moreover, all these TIME status results in the TCGA verification cohort and independent GEO verification cohort were similar, demonstrating that there were associations between the risk score and TIME status in patients with PTC (Figs. 5g-6f).

### Construction and calibration of a nomogram integrating the constructed risk model

A nomogram integrating risk score and clinicopathological features was constructed to predict the DFS of patients with PTC more precisely. The constructed nomogram showed the contribution of the risk score and clinicopathological features to the probability of DFS (Fig. 6g). The C-index of the nomogram in the training cohort reached 0.85 (95% CI: 0.78–0.92; *P* < 0.05). In addition, the AUCs of the ROC curve for 1-, 3-, and 5-year survival in the training cohort were 0.85, 0.88, and 0.85, respectively (Fig. 6h-i), and similar results were observed in the TCGA verification cohort (Fig. 6j-k). These results demonstrate that the integrated nomogram could accurately predict the DFS of patients with PTC.

### Expressional validation of four specific P-DECRGs

The expression levels of four specific P-DECRGs were compared between PTC and non-tumor thyroid tissues in the independent GEO verification cohort. In addition, the expression differences of four specific P-DECRGs between selected PTC and paracancerous thyroid tissues were also estimated by qPCR. The comparison results showed that the mRNA expression levels of *CDK1* and *P2RX4* in PTC tissues were significantly higher than those in non-tumor thyroid tissues. Moreover, the mRNA expression levels of *MAP1LC3A* and *FDX1* in PTC tissues were significantly lower than those in non-tumor thyroid tissues (Fig. 6l-m). The comparison results were consistent with those of the TCGA datasets.

## Discussion

Most PTCs are indolent, and patients have relatively favorable prognoses with a 10-year survival rate > 90% [25]. Because of the excellent overall survival (OS) of PTC, we paid more attention to another important endpoint (DFS) to evaluate its prognostic significance for patients with PTC in this study. Among the 476 patients with PTC from the TCGA database, the OS rate was 99.58% (474/476), while the DFS rate was 90.34% (430/476). For patients with PTC, DFS more accurately reflects disease status and the impact of the disease on the physical and mental states of patients than OS.

Tumor heterogeneity is a crucial phenomenon involved in the interactions among various factors, including certain intracellular genetic changes and tumor microenvironment influences [26]. As a result, tumor heterogeneity leads to the complexity of tumor cells and diversity in the therapeutic response [27]. Therefore, it is essential to administer specific treatment strategies based on the molecular subtype classification of PTC, in line with the concept of precision medicine [28]. Consensus clustering is a reliable approach in which several different clusters can be obtained to aggregate the clustering results and obtain a better clustering solution [29].

Accordingly, in this study, patients with PTC were divided into two different clusters, corresponding to two different molecular subtypes, using the consensus clustering method. Recent studies have suggested that copper is closely related to the occurrence and development of tumors [30]. Molecular subtype classification based on CRGs may reveal the exact molecular mechanisms and specific signaling pathways involved in tumor progression, TIME characteristics, and patient prognosis. A prognostic model for cuproptosis-related subtypes has been developed for glioma, providing new insights into tumor prognosis assessment, neoplasm-immune interactions, and potential drug targets [31]. Identifying cuproptosis-related subtypes and developing prognosis models in breast cancer are also useful predictors of prognosis and the tumor microenvironment [32]. In hepatocellular carcinoma, the CRG scoring model may inspire new approaches for both clinically predicting prognosis and developing treatment strategies [33]. However, the relationship between cuproptosis, PTC, and TIME status remains elusive. In the present study, patients with PTC were divided into clusters corresponding to two different molecular subtypes based on P-DECRGs. Further research indicated the diversity of prognosis and revealed the distinction of molecular mechanisms, especially those in immune regulatory mechanisms, between the two molecular subtypes. In addition, the constructed risk model was proven to be a well-established scoring system for predicting prognosis and evaluating the TIME status of each patient with PTC, which helped specify individualized treatment and follow-up strategy. Consequently, we speculate that cuproptosis plays an important role in tumor development, regulation of the TIME status, and prognosis in PTC.

Further bioinformatics analysis revealed the enrichment of many invasion-migration-associated pathways. These pathways, including biological adhesion, cell migration, locomotion, leukocyte migration, cell adhesion molecules, focal adhesion, and ECM-receptor interaction, have been widely confirmed to be associated with the prognosis of patients with PTC [34]. In addition, some immune-related pathways, such as the positive regulation of immune system processes and MHC protein complexes, were also enriched. Therefore, cuproptosis may inhibit the progression and improve the prognosis of PTC by regulating invasion-migration-associated pathways and mediating these immune regulation processes.

The tumor microenvironment consists primarily of several different types of immune cells, extracellular matrix, and stromal cells, which provide tumor cells with support and nourishment. Tumor biology highly depends on the tumor microenvironment, especially the immune microenvironment [35]. Copper, which is essential for the immune response, plays a vital role in both cellular and humoral immunity [36, 37]. Various immune cells, including B cells, T helper cells, macrophages, and NK cells, can be manipulated by copper to activate and maintain the immune system [38]. In the present study, multiple immune analysis algorithms were applied to comprehensively evaluate the relationship between TIME status and the P-DECRG-based subtypes and the risk model. The results showed that the TIME features, as well as the abundance of many immune infiltrating cells, varied substantially across the two distinct P-DECRG-based molecular subtypes and various P-DECRG risk scores. Although there were some inconsistencies between the results of different immune algorithms, the significantly different trend of Tregs was always observed. Tumor-infiltrating Tregs inhibit antitumor immunity and promote cancer progression, with poor clinical outcomes [39]. Recent studies have demonstrated that the CRG signature correlates with the infiltration of Tregs in osteosarcoma [40] and melanoma [41]. Therefore, cuproptosis may improve the response to immunotherapy by inhibiting Treg infiltration into the tumor microenvironment of PTC. Based on current knowledge, immunotherapy, which has witnessed breakthrough progress in recent years, has provided a new strategy for tumor treatment. Among the various types of immunotherapies, immune checkpoint blockade therapy aims to inhibit or block the interaction of certain immune checkpoints with their ligands [42]. Experimental and clinical studies of immune checkpoint inhibitors, including those targeting programmed cell death protein-1, programmed cell death-Ligand 1 (PD-L1), and cytotoxic T lymphocyte-associated antigen-4, have demonstrated improved treatment outcomes. Intra-tumoral copper supplementation enhances PD-L1 expression, affects the number of infiltrated CD8^+^ T and NK cells, and regulates key cancer immune evasion signaling pathways driven by PD-L1 [43]. In the present study, significant differences were observed in the receipt of immune checkpoint blockade therapy between these two subtypes, and immune checkpoint blockade therapy could be a favorable choice for treating certain PTC subtypes.

Genome-wide CRISPR-Cas9 loss-of-function screens have been reported, and 10 genes closely related to cuproptosis have been identified [9]. In the current study, we included a large number of genes, including 135 genes associated with copper homeostasis and transport, to comprehensively evaluate the influence of copper on the progression and prognosis of PTC. After stepwise screening and analysis, four specific P-DECRGs (*CDK1*, *FDX1*, *MAP1LC3A*, and *P2RX4*) related to the prognosis and TIME status of PTC were identified, which was not completely consistent with the results of a previously published study [44]. Several explanations, including differences in tumor types, may explain this discrepancy.

*FDX1* encodes a small iron-sulfur (Fe-S) protein that is present in the matrix of human mitochondria. In addition to its function in mitochondrial Fe-S cluster biogenesis, FDX1 is considered a versatile electron mediator involved in various physiological and pathological processes [45–47]. For example, in lung adenocarcinoma, *FDX1* has been reported to be closely involved in fatty acid oxidation, glucose, and amino acid metabolism [48]. However, in PTC, the functions and mechanisms of *FDX1* have not been established. Nevertheless, the results of this study indicate that *FDX1* may be negatively associated with the progression and prognosis of PTC through the mediation of cuproptosis.

*CDK1* encodes a serine/threonine kinase that functions as a cell cycle checkpoint protein and plays a key role in controlling eukaryotic cell cycle progression [49]. Binding to different cyclin proteins, CDK1 is considered the most critical cell cycle element and is sufficient to regulate numerous steps in cell proliferation and organ development [50]. Recently, the cell cycle-independent function of CDK1 in enhancing overall protein synthesis has been uncovered [51]. An integrative human pan-cancer analysis showed that dysregulation of CDK1 was identified in more than 20 human tumors and was significantly correlated with the development, progression, and microenvironment of tumor cells [52]. Low-dose radiation exposure alters the expression of CDK1, which is associated with post-Chernobyl PTC development [53]. In addition, CDK1 mediates the proliferation of PTC cells induced by high iodine [54]. Our results in this study were consistent with those of other studies. Currently, the role of *CDK1* in PTC progression remains unclear. We speculated that CDK1-related cuproptosis might be associated with energy metabolism reprogramming in PTC cells due to the vital function of CDK1 in regulating mitochondrial bioenergetics [55].

*P2RX4* encodes a receptor protein of the P2X4 ATP-gated nonselective ionotropic channel, which has emerged as a key molecule controlling the release of neuronal brain-derived neurotrophic factor and is involved in pain processing [56]. Emerging evidence has uncovered the role of *P2RX4* in cancer biology, including cancer pain [57]. In prostate cancer, *P2RX4* is involved in enhancing tumor formation, mobility, TGFβ-1 induced invasiveness, and epithelial-to-mesenchymal transition [58, 59]. In hepatocellular carcinoma, *P2RX4* is highly expressed and positively related to the growth and proliferation of cancer cells [60]. Moreover, *P2RX4* may play a vital role in regulating tumor development through inflammation and immune responses in the microenvironment [61]. In the present study, *P2RX4* expression was positively associated with the progression and prognosis of PTC by regulating the ionotropic channel related to cuproptosis.

Based on current knowledge, autophagy plays a crucial role in intracellular copper transportation [62]. Microtubule-associated protein 1A/1B light chain 3 (LC3) is a well-known biomarker for evaluating autophagy. Three LC3 proteins exist in humans: LC3A, LC3B, and LC3C [63]. Among these three members, *MAP1LC3A* encodes LC3A, which functions as a structural protein involved in mediating physical interactions between microtubules and other cytoskeleton elements. *MAP1LC3A* expression is downregulated in multiple tumors [64], and in the current study, *MAP1LC3A* expression was also suppressed in PTC, suggesting that its decreased expression may be related to the development of PTC cells. However, the exact molecular roles of *MAP1LC3A* in PTC pathogenesis require further study since the interactions between copper metabolism and autophagy are mostly unexplored.

The current study has some limitations. In the current study, no prognostic information on the selected patients with PTC was obtained due to the limited follow-up time. The number of patients with PTC selected for validation was small. Although the signatures of the four specific P-DECRGs were observed to be potential therapeutic targets or predictive biomarkers for PTC, the exact molecular mechanisms underlying their functions in cuproptosis have not yet been clarified. Therefore, further studies should include an expanded enrollment of cases and long-term follow-up to construct an independent validation cohort and confirm the predictive effect of the constructed risk model. In addition, further research is necessary to investigate the functions and molecular mechanisms of the four specific P-DECRGs in PTC.

## Conclusions

In summary, in the present study, we identified two PTC molecular subtypes based on CRGs via consensus clustering. We also constructed a prognostic related predictive risk model using the signature of the four specific P-DECRGs, which was closely associated with the TIME status of patients with PTC. Our findings may provide valuable clinical guidance for either prognostic prediction or the development of precision treatment strategies for patients with PTC.

## Supplementary Information

**Additional file 1: Table S1. **Details of 13 gene sets and overall unique cuproptosis related genes included in this study.

**Additional file 2: Table S2.** Details of 21 P-DECRGs generated from the results of Wilcoxon rank sum test and univariable Cox analysis in this study.

## Data Availability

The datasets used and/or analyzed during the current study are available from the corresponding author upon reasonable request.

## Abbreviations

CRGs: Cuproptosis-related genes
PTC: Papillary thyroid carcinoma
TCGA: The Cancer Genome Atlas
TIME: Tumor immune microenvironment
qPCR: Quantitative real-time polymerase chain reaction
DFS: Disease-free survival
OS: Overall survival
NA: Not available
MSigDB: Molecular Signatures Database
PCA: Principal component analysis
DEG: Differentially expressed genes
GO: Gene Ontology
KEGG: Kyoto Encyclopedia of Genes and Genomes
FDR: False discovery rate
ICB: Immune checkpoint blockade
LASSO: Least absolute shrinkage and selection operator
ROC: Receiver operating characteristic
AUC: Area under the curve
GAPDH: Glyceraldehyde-3-phosphate dehydrogenase
Tregs: Regulatory T cells
MHC: Major histocompatibility complex

## References

[1] Li M, Dal Maso L, Vaccarella S (2020). Global trends in thyroid cancer incidence and the impact of overdiagnosis. Lancet Diabetes Endocrinol.

[2] Li M, Brito JP, Vaccarella S (2020). Long-Term Declines of Thyroid Cancer Mortality: An International Age-Period-Cohort Analysis. Thyroid.

[3] Siegel RL, Miller KD, Fuchs HE, Jemal A (2021). Cancer Statistics, 2021. CA Cancer J Clin.

[4] Wang J, Yu F, Shang Y, Ping Z, Liu L (2020). Thyroid cancer: incidence and mortality trends in China, 2005–2015. Endocrine.

[5] Megwalu UC, Moon PK (2022). Thyroid Cancer Incidence and Mortality Trends in the United States: 2000–2018. Thyroid.

[6] Ito Y, Miyauchi A, Kihara M, Fukushima M, Higashiyama T, Miya A (2018). Overall Survival of Papillary Thyroid Carcinoma Patients: A Single-Institution Long-Term Follow-Up of 5897 Patients. World J Surg.

[7] Liu C, Xiao C, Chen J, Li X, Feng Z, Gao Q, Liu Z (2019). Risk factor analysis for predicting cervical lymph node metastasis in papillary thyroid carcinoma: a study of 966 patients. BMC Cancer.

[8] Babak MV, Ahn D (2021). Modulation of Intracellular Copper Levels as the Mechanism of Action of Anticancer Copper Complexes: Clinical Relevance. Biomedicines.

[9] Tsvetkov P, Coy S, Petrova B, Dreishpoon M, Verma A, Abdusamad M, Rossen J, Joesch-Cohen L, Humeidi R, Spangler RD (2022). Copper induces cell death by targeting lipoylated TCA cycle proteins. Science.

[10] Cerami E, Gao J, Dogrusoz U, Gross BE, Sumer SO, Aksoy BA, Jacobsen A, Byrne CJ, Heuer ML, Larsson E (2012). The cBio cancer genomics portal: an open platform for exploring multidimensional cancer genomics data. Cancer Discov.

[11] Gao J, Aksoy BA, Dogrusoz U, Dresdner G, Gross B, Sumer SO, Sun Y, Jacobsen A, Sinha R, Larsson E (2013). Integrative analysis of complexcancer genomics and clinical profiles using the cBioPortal. Sci Signal.

[12] Liberzon A, Subramania A, Pinchback R, Thorvaldsdottir H, Tamayo P, Mesirov JP (2011). Molecular signatures database (MSigDB) 3.0. Bioinformatics.

[13] Wilkerson MD, Hayes DN (2010). ConsensusClusterPlus: a class discovery tool with confidence assessments and item tracking. Bioinformatics.

[14] Ritchie ME, Phipson B, Wu D, Hu Y, Law CW, Shi W, Smyth GK (2015). limma powers differential expression analyses for RNA-sequencing and microarray studies. Nucleic Acids Res.

[15] Kanehisa M, Goto S (2000). KEGG: kyoto encyclopedia of genes and genomes. Nucleic Acids Res.

[16] Kanehisa M (2019). Toward understanding the origin and evolution of cellular organisms. Protein Sci.

[17] Kanehisa M, Furumichi M, Sato Y, Ishiguro-Watanabe M, Tanabe M (2021). KEGG: integrating viruses and cellular organisms. Nucleic Acids Res.

[18] Yoshihara K, Shahmoradgoli M, Martinez E, Vegesna R, Kim H, Torres-Garcia W, Trevino V, Shen H, Laird PW, Levine DA (2013). Inferring tumour purity and stromal and immune cell admixture from expression data. Nat Commun.

[19] Charoentong P, Finotello F, Angelova M, Mayer C, Efremova M, Rieder D, Hackl H, Trajanoski Z (2017). Pan-cancer Immunogenomic Analyses Reveal Genotype-Immunophenotype Relationships and Predictors of Response to Checkpoint Blockade. Cell Rep.

[20] Newman AM, Liu CL, Green MR, Gentles AJ, Feng W, Xu Y, Hoang CD, Diehn M, Alizadeh AA (2015). Robust enumeration of cell subsets from tissue expression profiles. Nat Methods.

[21] Finotello F, Mayer C, Plattner C, Laschober G, Rieder D, Hackl H, Krogsdam A, Loncova Z, Posch W, Wilflingseder D (2019). Molecular and pharmacological modulators of the tumor immune contexture revealed by deconvolution of RNA-seq data. Genome Med.

[22] Miao YR, Zhang Q, Lei Q, Luo M, Xie GY, Wang H, Guo AY (2020). ImmuCellAI: A Unique Method for Comprehensive T-Cell Subsets Abundance Prediction and its Application in Cancer Immunotherapy. Adv Sci (Weinh).

[23] Iasonos A, Schrag D, Raj GV, Panageas KS (2008). How to build and interpret a nomogram for cancer prognosis. J Clin Oncol.

[24] Wang L, Huang Y, Liu C, Guo M, Ma Z, He J, Wang A, Sun X, Liu Z (2021). Deltex3 inhibits Epithelial Mesenchymal Transition in Papillary Thyroid Carcinoma via promoting ubiquitination of XRCC5 to regulate the AKT signal pathway. J Cancer.

[25] Haugen BR, Alexander EK, Bible KC, Doherty GM, Mandel SJ, Nikiforov YE, Pacini F, Randolph GW, Sawka AM, Schlumberger M (2016). 2015 American Thyroid Association Management Guidelines for Adult Patients with Thyroid Nodules and Differentiated Thyroid Cancer: The American Thyroid Association Guidelines Task Force on Thyroid Nodules and Differentiated Thyroid Cancer. Thyroid.

[26] Pe'er D, Ogawa S, Elhanani O, Keren L, Oliver TG, Wedge D (2021). Tumor heterogeneity. Cancer Cell.

[27] Dagogo-Jack I, Shaw AT (2018). Tumour heterogeneity and resistance to cancer therapies. Nat Rev Clin Oncol.

[28] Gawin M, Kurczyk A, Stobiecka E, Fratczak K, Polanska J, Pietrowska M, Widlak P (2019). Molecular Heterogeneity of Papillary Thyroid Cancer: Comparison of Primary Tumors and Synchronous Metastases in Regional Lymph Nodes by Mass Spectrometry Imaging. Endocr Pathol.

[29] Li J, Xie L, Xie Y, Wang F (2020). Bregmannian consensus clustering for cancer subtypes analysis. Comput Methods Programs Biomed.

[30] Oliveri V (2022). Selective Targeting of Cancer Cells by Copper Ionophores: An Overview. Front Mol Biosci.

[31] Chen B, Zhou X, Yang L, Zhou H, Meng M, Zhang L, Li J (2022). A Cuproptosis Activation Scoring model predicts neoplasm-immunity interactions and personalized treatments in glioma. Comput Biol Med.

[32] Li Z, Zhang H, Wang X, Wang Q, Xue J, Shi Y, Wang M, Wang G, Zhang J (2022). Identification of cuproptosis-related subtypes, characterization of tumor microenvironment infiltration, and development of a prognosis model in breast cancer. Front Immunol.

[33] Zhang Z, Zeng X, Wu Y, Liu Y, Zhang X, Song Z (2022). Cuproptosis-Related Risk Score Predicts Prognosis and Characterizes the Tumor Microenvironment in Hepatocellular Carcinoma. Front Immunol.

[34] Reyes I, Reyes N, Suriano R, Iacob C, Suslina N, Policastro A, Moscatello A, Schantz S, Tiwari RK, Geliebter J (2019). Gene expression profiling identifies potential molecular markers of papillary thyroid carcinoma. Cancer Biomark.

[35] Pansy K, Uhl B, Krstic J, Szmyra M, Fechter K, Santiso A, Thuminger L, Greinix H, Kargl J, Prochazka K (2021). Immune Regulatory Processes of the Tumor Microenvironment under Malignant Conditions. Int J Mol Sci.

[36] Djoko KY, Ong CL, Walker MJ, McEwan AG (2015). The Role of Copper and Zinc Toxicity in Innate Immune Defense against Bacterial Pathogens. J Biol Chem.

[37] Hackler J, Heller RA, Sun Q, Schwarzer M, Diegmann J, Bachmann M, Moghaddam A, Schomburg L (2021). Relation of Serum Copper Status to Survival in COVID-19. Nutrients.

[38] Su Y, Zhang X, Li S, Xie W, Guo J (2022). Emerging Roles of the Copper-CTR1 Axis in Tumorigenesis. Mol Cancer Res.

[39] Goswami TK, Singh M, Dhawan M, Mitra S, Emran TB, Rabaan AA, Mutair AA, Alawi ZA, Alhumaid S, Dhama K (2022). Regulatory T cells (Tregs) and their therapeutic potential against autoimmune disorders - Advances and challenges. Hum Vaccin Immunother.

[40] Yang M, Zheng H, Xu K, Yuan Q, Aihaiti Y, Cai Y, Xu P (2022). A novel signature to guide osteosarcoma prognosis and immune microenvironment: Cuproptosis-related lncRNA. Front Immunol.

[41] Lv H, Liu X, Zeng X, Liu Y, Zhang C, Zhang Q, Xu J (2022). Comprehensive Analysis of Cuproptosis-Related Genes in Immune Infiltration and Prognosis in Melanoma. Front Pharmacol.

[42] Petitprez F, Meylan M, de Reynies A, Sautes-Fridman C, Fridman WH (2020). The Tumor Microenvironment in the Response to Immune Checkpoint Blockade Therapies. Front Immunol.

[43] Voli F, Valli E, Lerra L, Kimpton K, Saletta F, Giorgi FM, Mercatelli D, Rouaen JRC, Shen S, Murray JE (2020). Intratumoral Copper Modulates PD-L1 Expression and Influences Tumor Immune Evasion. Cancer Res.

[44] Bian Z, Fan R, Xie L (2022). A Novel Cuproptosis-Related Prognostic Gene Signature and Validation of Differential Expression in Clear Cell Renal Cell Carcinoma. Genes (Basel).

[45] Cai K, Tonelli M, Frederick RO, Markley JL (2017). Human Mitochondrial Ferredoxin 1 (FDX1) and Ferredoxin 2 (FDX2) Both Bind Cysteine Desulfurase and Donate Electrons for Iron-Sulfur Cluster Biosynthesis. Biochemistry.

[46] Wang Z, Dong H, Yang L, Yi P, Wang Q, Huang D (2021). The role of FDX1 in granulosa cell of Polycystic ovary syndrome (PCOS). BMC Endocr Disord.

[47] Tsvetkov P, Detappe A, Cai K, Keys HR, Brune Z, Ying W, Thiru P, Reidy M, Kugener G, Rossen J (2019). Mitochondrial metabolism promotes adaptation to proteotoxic stress. Nat Chem Biol.

[48] Zhang Z, Ma Y, Guo X, Du Y, Zhu Q, Wang X, Duan C (2021). FDX1 can Impact the Prognosis and Mediate the Metabolism of Lung Adenocarcinoma. Front Pharmacol.

[49] Xiang C, Sun WH, Ke Y, Yu X, Wang Y (2022). CDCA8 Contributes to the Development and Progression of Thyroid Cancer through Regulating CDK1. J Cancer.

[50] Bhattacharyya N, Gupta S, Sharma S, Soni A, Bagabir SA, Bhattacharyya M, Mukherjee A, Almalki AH, Alkhanani MF, Haque S (2022). CDK1 and HSP90AA1 Appear as the Novel Regulatory Genes in Non-Small Cell Lung Cancer: A Bioinformatics Approach. J Pers Med.

[51] Haneke K, Schott J, Lindner D, Hollensen AK, Damgaard CK, Mongis C, Knop M, Palm W, Ruggieri A, Stoecklin G (2020). CDK1 couples proliferation with protein synthesis. J Cell Biol.

[52] Liu X, Wu H, Liu Z (2022). An Integrative Human Pan-Cancer Analysis of Cyclin-Dependent Kinase 1 (CDK1). Cancers (Basel).

[53] Handkiewicz-Junak D, Swierniak M, Rusinek D, Oczko-Wojciechowska M, Dom G, Maenhaut C, Unger K, Detours V, Bogdanova T, Thomas G (2016). Gene signature of the post-Chernobyl papillary thyroid cancer. Eur J Nucl Med Mol Imaging.

[54] Lv C, Gao Y, Yao J, Li Y, Lou Q, Zhang M, Tian Q, Yang Y, Sun D (2021). High Iodine Induces the Proliferation of Papillary and Anaplastic Thyroid Cancer Cells via AKT/Wee1/CDK1 Axis. Front Oncol.

[55] Xie B, Wang S, Jiang N, Li JJ (2019). Cyclin B1/CDK1-regulated mitochondrial bioenergetics in cell cycle progression and tumor resistance. Cancer Lett.

[56] Lalisse S, Hua J, Lenoir M, Linck N, Rassendren F, Ulmann L (2018). Sensory neuronal P2RX4 receptors controls BDNF signaling in inflammatory pain. Sci Rep.

[57] Zhang WJ, Luo C, Pu FQ, Zhu JF, Zhu Z (2020). The role and pharmacological characteristics of ATP-gated ionotropic receptor P2X in cancer pain. Pharmacol Res.

[58] He J, Zhou Y, Arredondo Carrera HM, Sprules A, Neagu R, Zarkesh SA, Eaton C, Luo J, Gartland A, Wang N (2020). Inhibiting the P2X4 Receptor Suppresses Prostate Cancer Growth In Vitro and In Vivo, Suggesting a Potential Clinical Target. Cells.

[59] Ghalali A, Ye ZW, Hogberg J, Stenius U (2020). PTEN and PHLPP crosstalk in cancer cells and in TGFbeta-activated stem cells. Biomed Pharmacother.

[60] Asif A, Khalid M, Manzoor S, Ahmad H, Rehman AU (2019). Role of purinergic receptors in hepatobiliary carcinoma in Pakistani population: an approach towards proinflammatory role of P2X4 and P2X7 receptors. Purinergic Signal.

[61] Khalid M, Manzoor S, Ahmad H, Asif A, Bangash TA, Latif A, Jaleel S (2018). Purinoceptor expression in hepatocellular virus (HCV)-induced and non-HCV hepatocellular carcinoma: an insight into the proviral role of the P2X4 receptor. Mol Biol Rep.

[62] Jiang Y, Huo Z, Qi X, Zuo T, Wu Z (2022). Copper-induced tumor cell death mechanisms and antitumor theragnostic applications of copper complexes. Nanomedicine (Lond).

[63] Baeken MW, Weckmann K, Diefenthaler P, Schulte J, Yusifli K, Moosmann B, Behl C, Hajieva P (2020). Novel Insights into the Cellular Localization and Regulation of the Autophagosomal Proteins LC3A, LC3B and LC3C. Cells.

[64] Wang Z, Gao L, Guo X, Feng C, Lian W, Deng K, Xing B (2019). Development and validation of a nomogram with an autophagy-related gene signature for predicting survival in patients with glioblastoma. Aging (Albany NY).

