# Supplementary material for: Construction of a novel cuproptosis-related gene signature for predicting prognosis and estimating tumor immune microenvironment status in papillary thyroid carcinoma
Source: BMC Cancer. 2022 Nov 4;22:1131. doi: 10.1186/s12885-022-10175-5 (PMC9635208; doi:10.1186/s12885-022-10175-5)
Supplement: Supplementary file 1 — Additional file 1: Table S1. Details of 13 gene sets and overall unique cuproptosis related genes included in this study. [file 12885_2022_10175_MOESM1_ESM.docx]

**Table S1 Details of 13 gene sets and overall unique cuproptosis related genes included in this study.**

| No. | Gene sets | Details |
| --- | --- | --- |
| 1 | WP_COPPER_HOMEOSTASIS | COX17 ADAM10 SLC31A1 SLC31A2 COX11 COMMD1 AKT1 MTF2 FOXO1 FOXO3 BACE1 STEAP2 STEAP1 GSK3B APC XIAP APP JUN MAPT MDM2 MT1A MT1B MT1E MT1F MT1G MT1H MT1JP MT1L MT1X MT2A MT3 MTF1 ATOX1 SLC11A2 PIK3CA ATP7A ATP7B XAF1 STEAP3 PRNP PTEN CCND1 SCO1 SOD1 SOD3 SP1 ADAM17 TP53 STEAP4 CASP3 MT4 ADAM9 CCS SCO2 |
| 2 | GOBP_RESPONSE_TO_COPPER_ION | PARK7 AANAT CYP1A1 DAXX BACE1 AOC1 MT1DP HSF1 APP IL1A AQP1 AQP2 LCAT LOXL2 MT1A MT1B MT1E MT1F MT1G MT1H MT1M MT1X MT2A MT3 MT-CO1 NFE2L2 PAM ATP5F1D ATP7A ATP7B PRNP MT1HL1 SNCA SOD1 SOD3 SORD TFRC MAP1LC3A MT4 BECN1 CDK1 |
| 3 | GOBP_DETOXIFICATION_OF_COPPER_ION | PARK7 MT1DP MT1A MT1B MT1E MT1F MT1G MT1H MT1M MT1X MT2A MT3 ATP7A MT1HL1 MT4 |
| 4 | GOBP_COPPER_ION_TRANSPORT | COX17 SLC31A1 SLC31A2 CP FKBP4 STEAP2 HEPHL1 ATOX1 SLC11A2 CUTC ATP7A ATP7B STEAP3 STEAP4 MMGT1 HEPH |
| 5 | GOBP_COPPER_ION_TRANSMEMBRANE_TRANSPORT | SLC31A1 SLC31A2 STEAP2 SLC11A2 ATP7A ATP7B |
| 6 | GOBP_COPPER_ION_IMPORT | SLC31A1 STEAP2 ATP7A ATP7B STEAP3 STEAP4 |
| 7 | GOBP_COPPER_ION_HOMEOSTASIS | ABCB6 ANKRD9 SLC31A1 SLC31A2 COMMD1 PRND CCDC22 XIAP APP ARF1 MT2A ATOX1 CUTC ATP7A ATP7B PRNP SCO1 COX19 SCO2 |
| 8 | GOBP_CELLULAR_RESPONSE_TO_COPPER_ION | CYP1A1 DAXX BACE1 AOC1 MT1DP HSF1 APP AQP1 AQP2 MT1A MT1B MT1E MT1F MT1G MT1H MT1M MT1X MT2A MT3 NFE2L2 ATP7A PRNP MT1HL1 SNCA MAP1LC3A MT4 BECN1 |
| 9 | GOBP_CELLULAR_COPPER_ION_HOMEOSTASIS | ABCB6 ANKRD9 SLC31A1 SLC31A2 PRND CCDC22 APP ARF1 MT2A ATOX1 ATP7A ATP7B PRNP SCO1 COX19 SCO2 |
| 10 | GOMF_COPPER_ION_TRANSMEMBRANE_TRANSPORTER_ACTIVITY | SLC31A1 SLC31A2 SLC11A2 ATP7A ATP7B |
| 11 | GOMF_COPPER_CHAPERONE_ACTIVITY | COX17 PARK7 ATOX1 ATP7A SCO1 SCO2 |
| 12 | GOMF_COPPER_ION_BINDING | MOXD2P COX17 MTCO2P12 PARK7 COX11 CP LACC1 COMMD1 DBH DCT ALB F5 F8 OR5AR1 ADNP ATP13A2 PRND AOC1 MOXD1 GPC1 ANG SUMF1 AOC2 SNAI3 APOA4 HEPHL1 IL1A COA6 LOX LOXL1 LOXL2 MT3 MT-CO2 ATOX1 ACR P2RX4 PAM CUTC CUTA ATP7A ATP7B PRNP HAMP S100A5 S100A12 S100A13 SCO1 SNCB SNCA SNCG SOD1 SOD3 TP53 TYR LOXL4 LOXL3 AOC3 RNF7 HEPH CCS SCO2 |
| 13 | A published article (PMID: 35298263) | FDX1 LIAS LIPT1 DLD DLAT PDHA1 PDHB MTF1 GLS CDKN2A |
| 14 | Overall unique CRGs | ATP7A ATP7B SLC31A1 MT2A PRNP SLC31A2 ATOX1 MT3 APP SCO2 SCO1 MT1G MT1B MT1F MT1X MT1E MT4 MT1H MT1A STEAP2 SLC11A2 COX17 PARK7 BACE1 SOD3 SOD1 STEAP3 STEAP4 COMMD1 MT1DP MT1M MT1HL1 SNCA AOC1 CUTC PRND XIAP TP53 CCS COX11 MTF1 BECN1 MAP1LC3A AQP2 CYP1A1 DAXX HSF1 NFE2L2 AQP1 PAM IL1A LOXL2 HEPH CP HEPHL1 COX19 ARF1 ANKRD9 ABCB6 CCDC22 MTF2 PTEN FOXO3 APC JUN ADAM17 AKT1 GSK3B FOXO1 XAF1 MT1JP ADAM9 MAPT MDM2 ADAM10 SP1 MT1L CASP3 CCND1 PIK3CA STEAP1 ATP5F1D CDK1 TFRC SORD AANAT MT-CO1 LCAT MMGT1 FKBP4 MTCO2P12 ALB TYR HAMP S100A12 OR5AR1 AOC3 COA6 ANG MOXD1 S100A5 LOXL4 LOX RNF7 LACC1 DCT MT-CO2 F5 P2RX4 CUTA F8 DBH SNCG MOXD2P ACR SNAI3 ATP13A2 SUMF1 GPC1 S100A13 LOXL3 AOC2 SNCB ADNP LOXL1 APOA4 PDHA1 GLS DLAT CDKN2A LIPT1 PDHB DLD LIAS FDX1 |
